# Interleukin-17A promotes tongue squamous cell carcinoma metastasis through activating miR-23b/versican pathway

**DOI:** 10.18632/oncotarget.14255

**Published:** 2016-12-27

**Authors:** Tai Wei, Xin Cong, Xiang-Ting Wang, Xiao-Jian Xu, Sai-Nan Min, Peng Ye, Xin Peng, Li-Ling Wu, Guang-Yan Yu

**Affiliations:** ^1^ Department of Oral and Maxillofacial Surgery, Peking University School and Hospital of Stomatology, National Engineering Laboratory for Digital and Material Technology of Stomatology, and Beijing Key Laboratory of Digital Stomatology, Beijing, China; ^2^ Department of Physiology and Pathophysiology, Peking University School of Basic Medical Sciences, Key Laboratory of Molecular Cardiovascular Sciences, Ministry of Education, and Beijing Key Laboratory of Cardiovascular Receptors Research, Beijing, China; ^3^ Department of Cell and Developmental Biology, School of Life Science, University of Science and Technology of China, Hefei, China; ^4^ Department of Ophthalmology, Peking University Third Hospital, Beijing, China

**Keywords:** interleukin-17A, miR-23b, versican, tongue squamous cell carcinoma, metastasis

## Abstract

Interleukin-17A (IL-17A), a proinflammatory cytokine mainly produced by T helper 17 cells, exerts protumor or antitumor effects in different cancer entities. However, the exact role of IL-17A in carcinogenesis and progression of tongue squamous cell carcinoma (TSCC) remains unclear. Here, we found that the levels of IL-17A in serum and tumor samples were significantly increased in TSCC patients and positively correlated with tumor metastasis and clinical stage. Besides, IL-17A enhanced cell migration and invasion in SCC15, a TSCC cell line. Furthermore, IL-17A inversely correlated with miR-23b expression in TSCC specimens. *In vitro*, NF-κB inhibited miR-23b transcription by directly binding to its promoter region. IL-17A downregulated miR-23b expression via activating NF-κB signaling pathway characterized by increasing p65 expression in the nuclear and elevating the levels of p-IKKα and p-IκBα. Overexpression of miR-23b inhibited, whereas knockdown of miR-23b promoted migration and invasion abilities of SCC15 cells. Moreover, extracellular matrix protein versican was proved to be the direct target of miR-23b through luciferase assay. IL-17A increased versican levels *in vitro* and knockdown of versican by siRNA inhibited SCC15 cell migration and invasion. Taken together, these results reveal a novel mechanism that IL-17A in TSCC microenvironment promotes the migration and invasion of TSCC cells through targeting miR-23b/versican pathway.

## INTRODUCTION

Tumor microenvironment consists of multiple cells (fibroblasts, immune cells, etc) and extracellular elements (chemokines, cytokines, extracellular matrix, etc), which forms the chronic inflammatory stroma promoting tumor initiation and progression [1]. Multiple immune cells can interact with tumor cells through direct contact or by secreting various cytokines and chemokines, which facilitate tumor development in the cancerous pathological context [1]. Among those mediators, interleukin-17 (IL-17), the signature cytokine of T helper 17 (Th17) cells, has attracted much more attention. IL-17 family has six members including IL-17A, IL-17B, IL-17C, IL-17D, IL-17E and IL-17F [2]. Among them, IL-17E promotes the proliferation of breast cancer cells [3], whereas exerts antitumor effect towards human melanoma xenografts in mice [4]. IL-17F has been characterized in different cancer entities. For example, IL-17F is significantly elevated in prostate cancer and triggers increased NF-κB signaling in B and T cells in patients with chronic lymphocytic leukemia [5, 6]. However, the roles of IL-17B, IL-17C, and IL-17D in cancer are less defined. As a proinflammatory cytokine of Th17 cells secreted, IL-17A is the most active one compared with other five members in circulation [2]. IL-17A exerts diverse functions in promoting host defense against infection and contributes to autoimmune diseases including multiple sclerosis [7] and rheumatoid arthritis [8]. Moreover, IL-17A has been detected in multiple cancer entities, such as ovarian cancer [9] and pancreatic cancer [10]. The roles of IL-17A in cancers are controversial since both pro- and antitumor effects have been reported. IL-17A potentiates metastasis of hepatocellular carcinoma cells [11] and increases tumor resistance to vascular endothelial growth factor (VEGF) inhibition therapy, which makes it hard to treat certain types of cancers [12]. However, high expression of IL-17 is associated with improved progression-free survival in advanced ovarian cancer [13]. Animal experiment indicates that IL-17 overexpression in tumor cells suppresses tumor progression through enhanced antitumor immunity in immunocompetent mice [14]. Therefore, to clarify the exact role of IL-17A in cancer biology is very important.

Tongue squamous cell carcinoma (TSCC) represents the most frequent type of oral squamous cell carcinoma comprising around 41% [15]. High malignant degree combined with high rate of proliferation and early nodal metastasis are the main characteristics of TSCC [16]. Although encouraging advances in the treatment of TSCC have been achieved in recent years, it is still difficult to control the metastasis issue effectively and thus results in the unfavorable prognosis. TSCC is reported to derive from precancerous lesions which are exposed to chronic carcinogens including tobacco, alcohol, and betel quid chewing [17–19]. The continuous inflammatory status is associated with TSCC progression and the acquisition of an invasive phenotype. Studies have reported that non-steroidal anti-inflammatory drugs decrease the incidence and mortality of certain cancers (such as glioma and colon cancer) [20, 21]. Therefore, exploring the role of IL-17A in TSCC and its underlying mechanisms would be useful to understand the crosstalk between TSCC cells and tumor microenvironment, shedding new insights on TSCC prevention and treatment by targeting critical immuno-modulatory cytokine.

MicroRNAs (miRNAs) are small non-coding RNAs (21-25 nucleotides) that have been reported to negatively regulate gene expression at posttranscriptional level [22]. Numbers of studies have shown that microRNAs are important players during TSCC development, for example, miR-26a inhibits cell proliferation and cell cycle progression in TSCC [23] and miR-34a suppresses TSCC cell migration and invasion via targeting matrix metalloproteinase 9 and 14 [24]. Recent studies indicate that miR-23b is downregulated in multiple tumor types and serves as tumor-suppressor [25–27]. Moreover, IL-17 is confirmed to potentiate proinflammatory cytokines secretion during autoimmune pathogenesis by downregulating miR-23b expression. Overexpression of miR-23b suppresses IL-17 induced inflammatory cytokines expression by repressing the transcription of TGF-β activated kinase1/MAP3K7 binding protein 2 (Tab 2), Tab 3 and inhibitor of nuclear factor κ-B (NF-κB) kinase subunit α (IKK-α) [28]. Since miR-23b acts as an oncosuppressor in types of cancers and regulates the IL-17-mediated proinflammatory signaling pathways in autoimmune disease, it is meaningful to reveal the role of miR-23b in TSCC development and the regulatory relationship between IL-17A and miR-23b.

**Table 2:** Primers used for PCR analysis

Genes	Forward and reverse primers	Size (bp)
IL-17A	5’-ATTGGTGTCACTGCTACTGCTGC-3’5’-TGGTATTCCGGTTATGGATGTTC-3’	148
IL-17RA	5’-CGTCACACTCACTCTACGCAACCTT-3’5’-GCCCGTGATGAACCAGTACACC-3’	187
GAPDH	5’-ACATCATCCCTGCCTCTACTG-3’5’-CCTGCTTCACCACCTTCTTG-3’	180

In the present study, we investigated the role of IL-17A in TSCC patients and SCC15 cells (TSCC cell line) and explored its underlying mechanism. We found that increased IL-17A in local microenvironment plays a crucial role in accelerating TSCC progression. Moreover, we identified that IL-17A-induced miR-23b downregulation and versican upregulation is a key pathway to promote migration and invasion abilities of SCC15 cells.

## RESULTS

### Patient characteristics

The clinical characteristics of the 76 study subjects with TSCC and 15 healthy controls were displayed in Table 1. Briefly, the included TSCC group was composed of 53.9% males and 46.1% females; 42.1% of the cases were aged more than 60. Pathologically, 77.6% of the patients were classified as T1 and T2 stage, and 22.4% as T3 and T4 stage. Lymph node metastasis was involved in 44.7% of the patients.

**Table 1:** Basic characteristics of the TSCC patients and healthy controls

	Healthy controls(n = 15)	TSCC patients(n = 76)
Gender		
Male	6	41
Female	9	35
Age(mean ± SD years)	55.46 ± 11.03	55.47 ± 8.30
Tumor size		
T1-T2		59
T3-T4		17
Node metastasis		
Yes (N1 or N2)		34
No (N0)		42
Differentiation		
Well		37
Moderate		31
Poor		8
Clinical stage		
Early stage (I-II)		38
Advanced stage (III-IV)		38

### Serum IL-17A levels are elevated in TSCC patients

**Figure 1:**
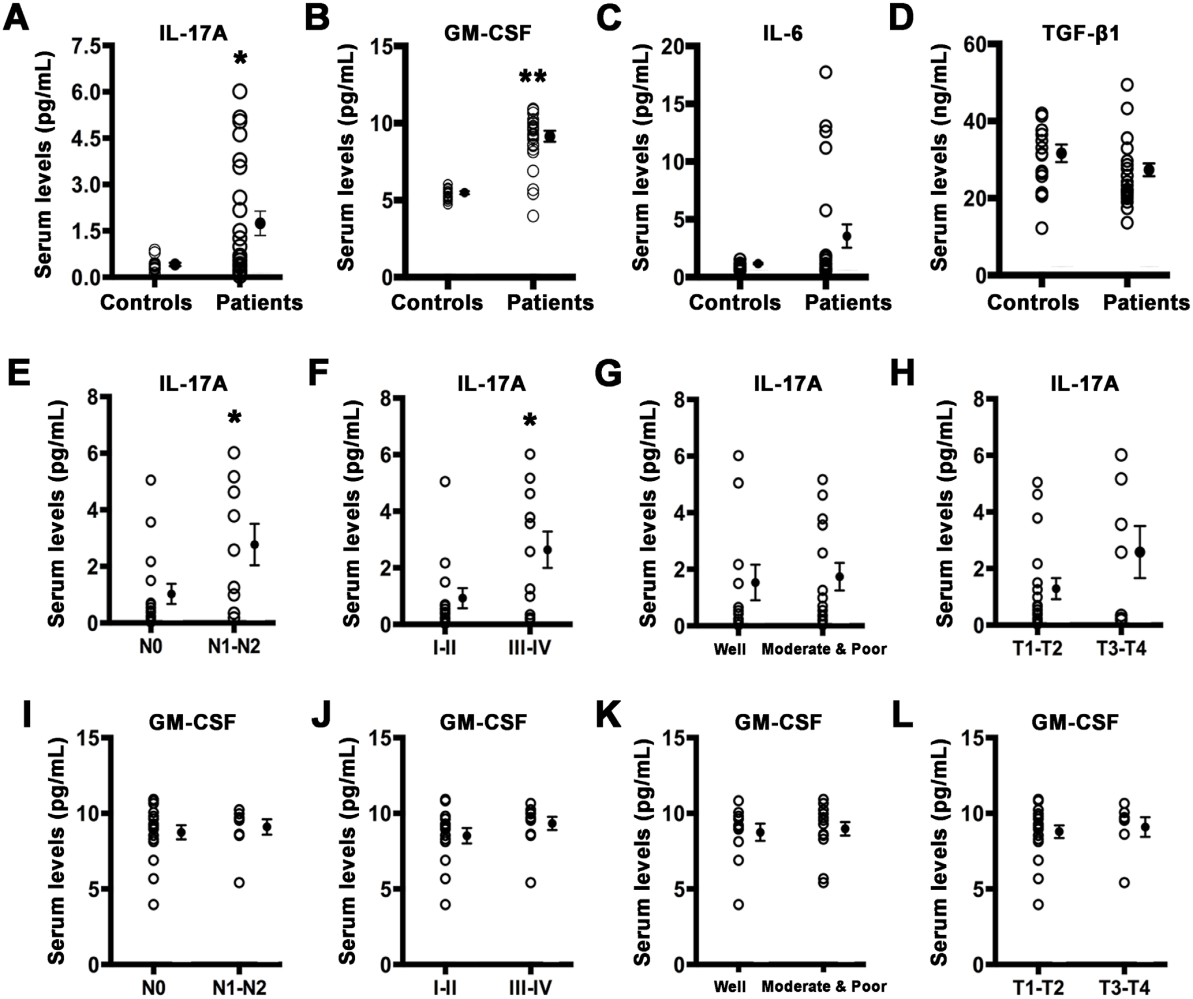
Serum IL-17A and GM-CSF are elevated in TSCC patients Serum levels of IL-17A **A**. GM-CSF **B**. IL-6 **C**. and TGF-β1 **D**. in healthy controls and TSCC patients. **E**. Serum IL-17A levels in TSCC patients with or without lymph node metastasis. **F**. Serum IL-17A levels in TSCC patients in early or advanced stage. **G**. Serum IL-17A levels in TSCC patients of different histological grade. **H**. Serum IL-17A levels in TSCC patients of different tumor size. **I**. Serum GM-CSF levels in TSCC patients with or without lymph node metastasis. **J**. Serum GM-CSF levels in TSCC patients in early or advanced stage. **K**. Serum GM-CSF levels in TSCC patients of different histological grade. **L**. Serum GM-CSF levels in TSCC patients of different tumor size. Open circles represent each subject and vertical lines indicate mean ± SEM. *P < 0.05 and **P < 0.01 compared with controls. N0: no regional lymph node metastasis; N1-N2: metastasis in a single, multiple ipsilateral or bilateral lymph node. T1: < 2 cm; T2: > 2, < 4 cm; T3: > 4 cm; T4: massive tumor which invades adjacent structures.

To evaluate the inflammatory status of TSCC patients, we detected a set of IL-17-associated cytokines levels in the serum of patients with TSCC and healthy controls. As presented in Figure 1, the levels of serum IL-17A and granulocyte macrophage colony stimulating factor (GM-CSF), one of the most common IL-17A-induced downstream cytokines, were significantly increased (Figure 1A and 1B) in TSCC patients compared with those in healthy controls. But no significant differences were observed in serum IL-6 and TGF-β1 between TSCC patients and healthy controls (Figure 1C and 1D). Moreover, serum IL-17A levels were higher in patients with lymph node metastasis or advanced clinical stages (Figure 1E and 1F), but showed no significant differences in histological grade and tumor size (Figure 1G and 1H). Although the level of serum IL-17A in male patientswas higher than that of females, there is no statistical difference between two groups (1.98 ± 0.58 pg/ml vs. 1.30 ± 0.49 pg/ml, *P* = 0.38). Serum GM-CSF levels did not correlated with the tested clinical characteristics (Figure 1I to 1L).

### IL-17A is overexpressed in TSCC and correlates with cancer progression

**Figure 2:**
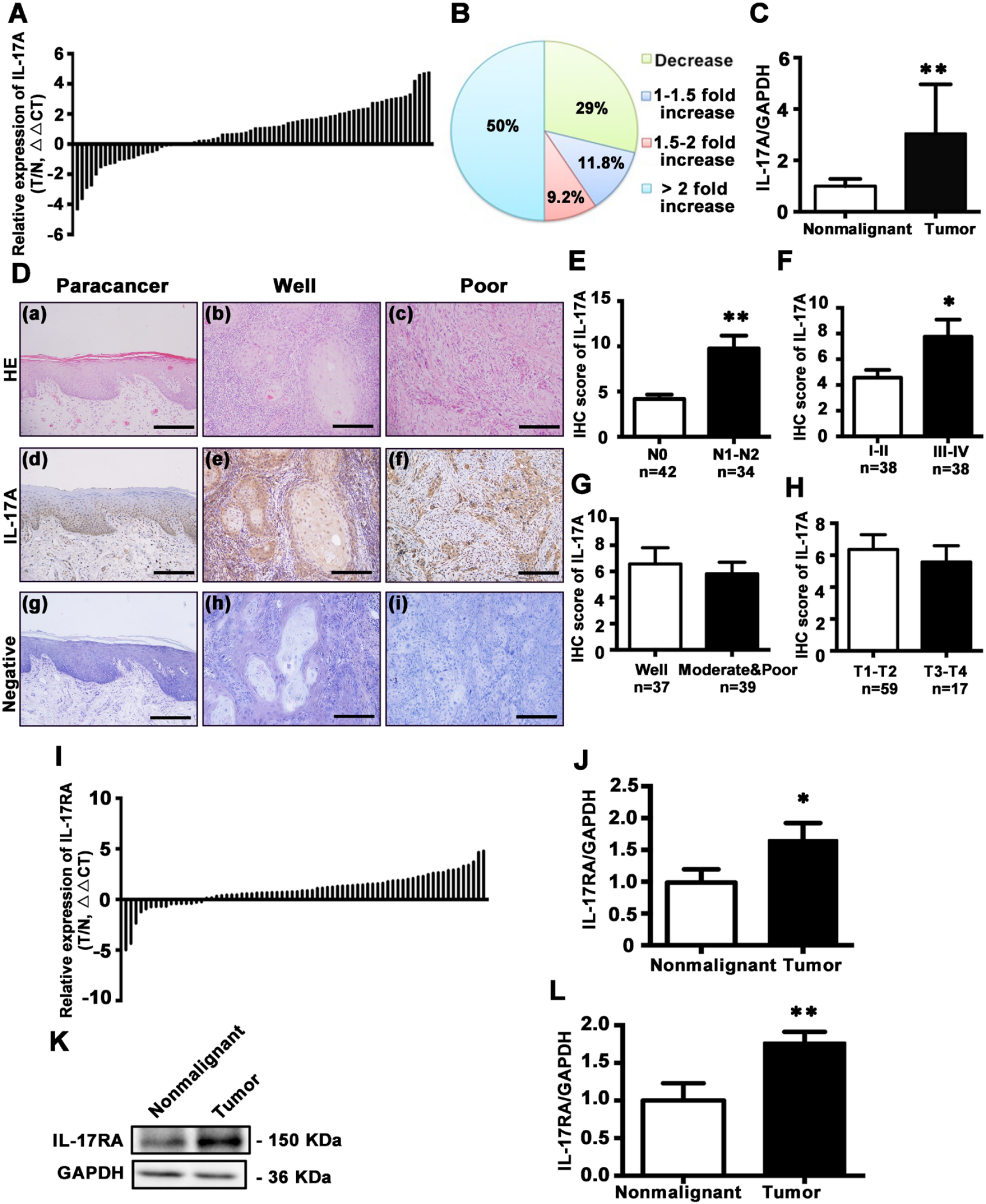
Expression and distribution of IL-17A and its receptor IL-17RA in tongue tissues **A**. Relative expression of IL-17A in 76 pairs of TSCC specimens and adjacent nonmalignant tissues was examined by qRT-PCR. Data were presented as relative fold change (ΔΔCT values, TSCC/Nonmalignant). **B**. IL-17A expression levels from tumors were normalized to their corresponding control and the percentage of cases for the indicated fold expression in the tumor represented as a pie chart. **C**. Relative IL-17A levels for 76 specimens of TSCC and its counterparts. Data were presented as relative mRNA fold change. **D**. Hematoxylin and eosin staining for paracancerous epithelium (a), well- (b), and poor-differentiated tumors (c). Immunohistochemical staining with anti-IL-17A antibody to characterize the expression and distribution of IL-17A in paracancerous epithelium (d), well- (e) and poor-differentiated tumors (f). Nuclei were counterstained with hematoxylin (blue). Primary antibody was omitted in negative controls (g, h, i). Scale bars: 200 μm. **E**. IHC scores of IL-17A expression based on lymph node metastasis. **F**. IHC scores of IL-17A expression based on clinical stages. **G**. IHC scores of IL-17A expression based on TSCC histological grade. **H**. IHC scores of IL-17A expression based on tumor size. **I**. Expression of IL-17RA in 72 pairs of TSCC and adjacent nonmalignant tissues was quantified by qRT-PCR. Data were presented as relative fold change (ΔΔCT values). **J**. Relative mRNA change of IL-17RA in TSCC and its counterparts. **K**. Representative western blot of IL-17RA in TSCC tissues and its counterparts. **L**. Quantitative analysis of IL-17RA protein expression based on western blot analysis of 36 pairs of TSCC and paracancer tissues. *P < 0.05, **P < 0.01.

To further establish association between IL-17A and TSCC risk, 76 pairs of TSCC and the adjacent histologically nonmalignant tissues were examined for IL-17A expression. IL-17A mRNA was significantly increased in 54 of 76 (71.0%) tumor samples compared with matched nonmalignant tissues (Figure 2A, and 2C, P < 0.01). Immunohistochemical staining showed that IL-17A was mainly localized in the stratum basale and stratum spinosum of normal epithelium, with slight expression in stratum granulosum and the hyper-orthokeratotic layer. In well-differentiated TSCC, IL-17A was mainly expressed in the basolateral cells around the keratin pearl, while in poorly-differentiated TSCC, it was widely expressed in almost all the tumor cells (Figure 2D). After quantification of immunohistochemical stainings of 76 patients, we found that the expressions of IL-17A were higher in patients with lymph node metastasis of N1 and N2 stages [29] than N0 (without metastasis) stage (Figure 2E). Moreover, the higher expression of IL-17A correlated with advanced clinical stages (stage III-IV) (Figure 2F), but did not differ with histological grade and tumor size (Figure 2G and 2H). In the 22 patients with decreased IL-17A levels, 60% of them (13/22) were I-II stage and N0 stage, 73% of them (16/22) were T1-T2 stage, and 82% of them (18/22) were well- or moderately-differentiated.

Next, we explored the expression of IL-17 receptor A (IL-17RA), the main receptor of IL-17A, in TSCC and nonmalignant tissues. The mRNA expression of IL-17RA was increased in 56 of 72 (77.8%) tumor samples compared with its matched counterparts (Figure 2I and 2J; *P* < 0.01). Western blot analysis also confirmed that IL-17RA expression was elevated in TSCC tissues (Figure 2K and 2L).

### IL-17A promotes cell mobility in SCC15 cells

**Figure 3:**
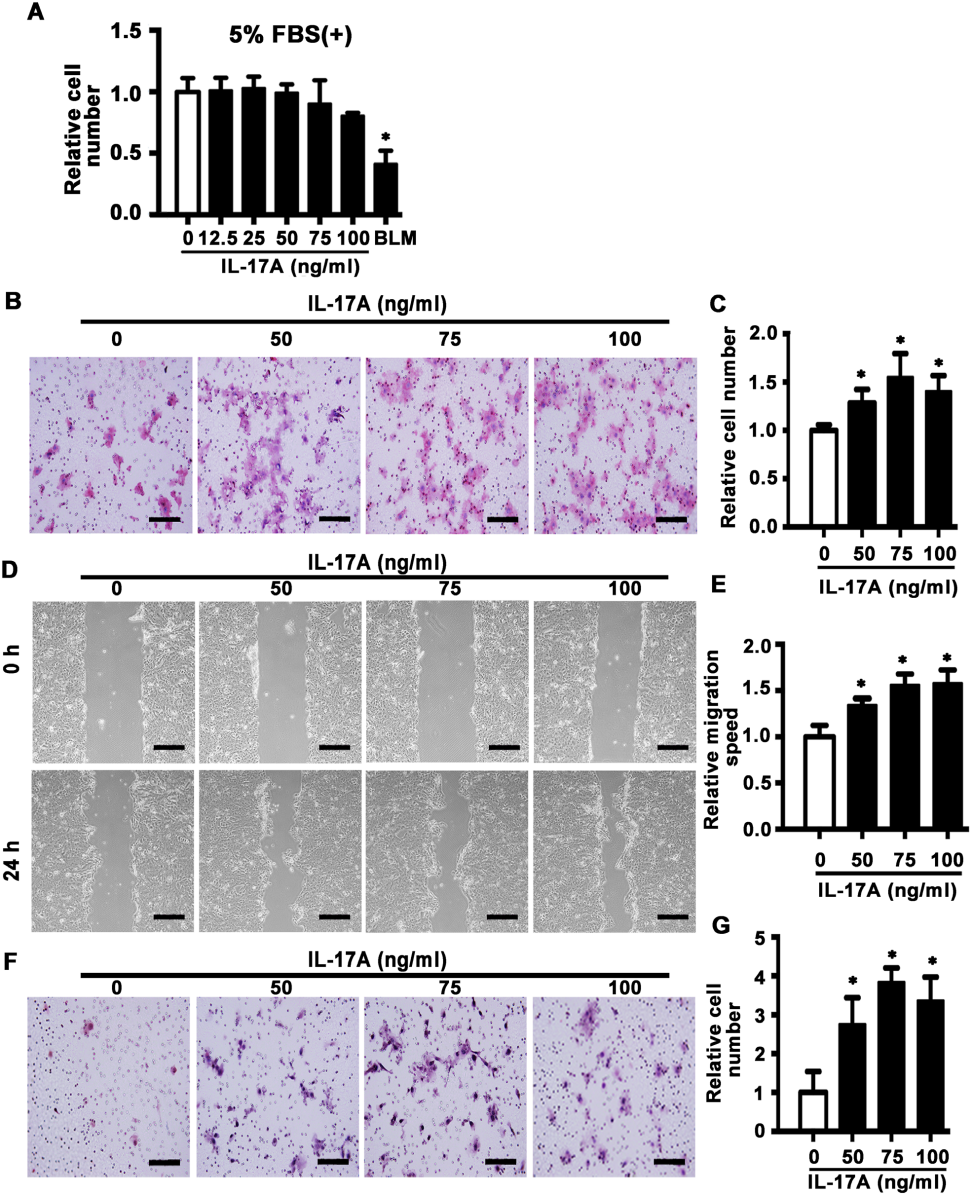
IL-17A promotes cell migration and invasion of SCC15 cells **A**. SCC15 cells were cultured with IL-17A from 12.5 to 100 ng/ml. The proliferation rate was detected by MTS assay 48 h after treatment. Bleomycin (BLM, 10 μg/ml) was used as positive control. **B**. Representative images of cell migration at 24 h. **C**. Histogram showing cell migration expressed as relative number of the whole migratory cells on the bottom side of the membrane. **D**. Representative images of wound-healing assay at 0 and 24 h. **E**. Histogram showing cell migration expressed as relative wound edges movement. **F**. Representative images of cell invasion at 24 h. **G**. Histogram showing cell invasion expressed as relative number of the whole invaded cells on the bottom side of the membrane. Values are presented as mean ± SEM from three independent experiments. Scale bars: 200 μm, *P < 0.05 compared with the untreated cells.

Next, we detected the effect of IL-17A on cell mobility by use of SCC15 cell line. MTS assay showed that IL-17A (12.5-100 ng/ml) had no effect on proliferation of SCC15 cells induced by 5% FBS for 48 h. Bleomycin (BLM), which is reported to inhibit proliferation of cancer cells [30], was used as positive control (Figure 3A). Transwell migration assay indicated that IL-17A (50, 75 and 100 ng/ml) incubation for 24 h enhanced the migration rate of SCC15 cells compared with control group (Figure 3B and 3C), which was further confirmed by wound healing assay (Figure 3D and 3E). In addition, invasive assay showed that IL-17A promoted the invasive ability of SCC15 cells (Figure 3F and 3G). These data indicate that increased IL-17A directly enhances the migration and invasive abilities of SCC15 cells.

### MiR-23b is downregulated in TSCC and inversely correlates with IL-17A expression in TSCC patients

**Figure 4:**
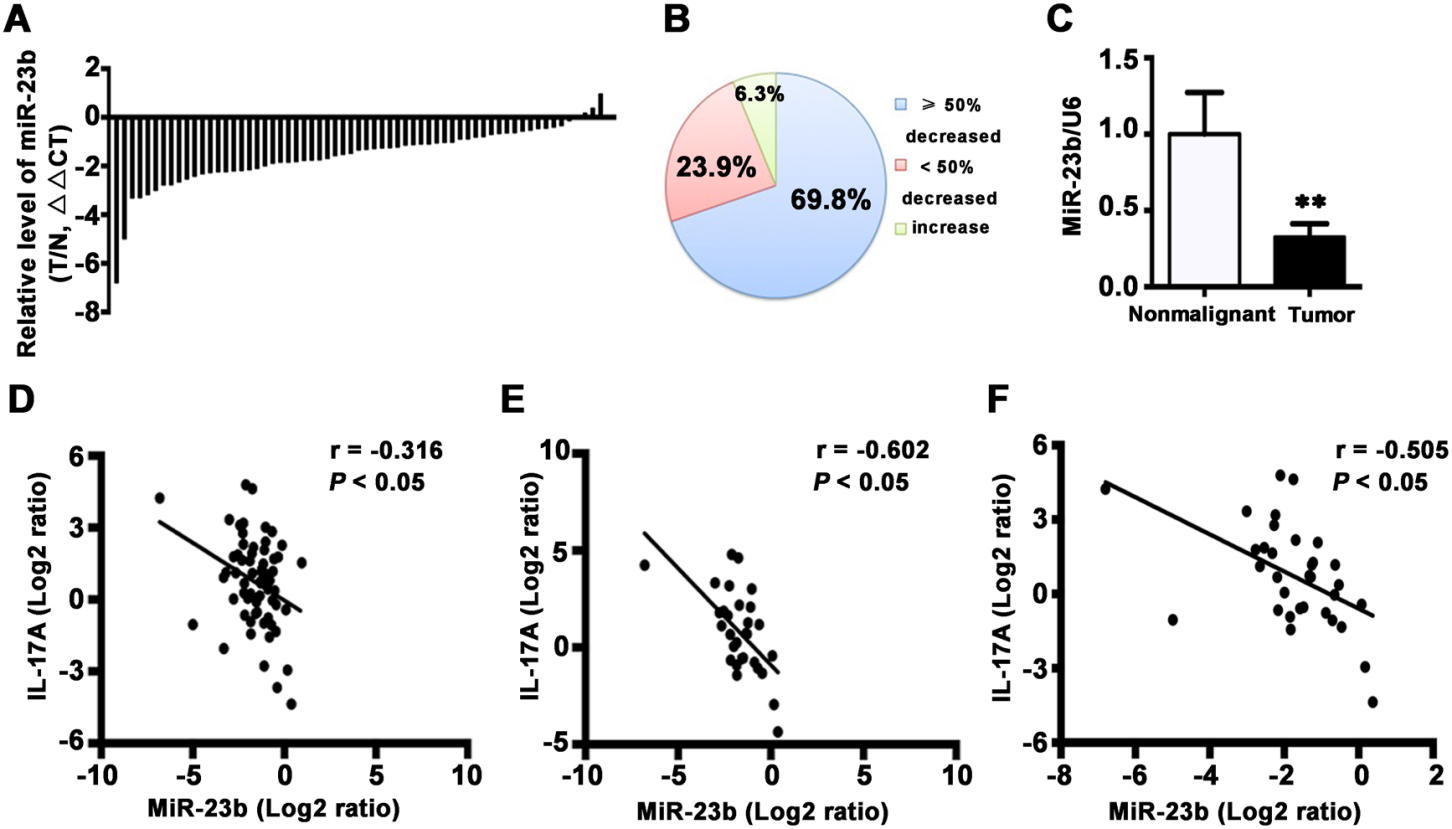
MiR-23b is decreased in TSCC specimens **A**. Relative levels of miR-23b in 63 TSCC specimens and adjacent nonmalignant tissues. Data were presented as relative fold change (ΔΔCT values). **B**. MiR-23b expression levels from tumors were normalized to their corresponding control and the percentage of cases for the indicated expression in the tumor represented as a pie chart. **C**. Relative miR-23b levels for 63 specimens of TSCC and its counterparts. Data were presented as relative mRNA fold change. **P < 0.01. The correlation between IL-17A and miR-23b expression in 63 pairs of TSCC samples **D**. in 30 paired specimens of TSCC patients with cervical metastasis **E**. and in 33 paired specimens of TSCC patients with advanced stages **F**. Values were presented with linear regression line and analyzed with Pearson's correlation method.

IL-17 is crucial for the downregulation of miR-23b in the pathogenesis of autoimmune disease [28]. To investigate whether IL-17 inhibited miR-23b expression contributes to TSCC progression, we examined miR-23b level in 63 pairs of TSCC and the adjacent nonmalignant tissues. MiR-23b was decreased in 59 of 63 (93.7%) cancer samples compared with the matched nonmalignant tissues (Figure 4A and 4B). Besides, the average expression level of miR-23b was significantly reduced in TSCC tissues (Figure 4C; *P* < 0.01). Moreover, a concomitant increase of IL-17A and decrease of miR-23b was observed in 66.7% of the patients (42/63) and IL-17A decrease coupled with miR-23b upregulation was observed in 4.8% of the samples (3/63). Pearson's correlation coefficient analysis revealed that expression of IL-17A was reversely correlated with expression of miR-23b (*r* = -0.316; *P* < 0.05) in all 63 tongue cancer samples (Figure 4D). This relationship became even more obvious when the analysis was restricted to the 30 tumor specimens with cervical metastasis (*r* = -0.602; *P* < 0.05) or the 33 advanced tumors (*r* = -0.505; *P* < 0.05) (Figure 4E and 4F). However, the correlation was not significant in 33 TSCC patients without lymph node metastasis (*r* = 0.099) or 30 early stage tumors (*r* = 0.039). These results suggest a negative regulatory role of IL-17A in miR-23b expression and it is likely to be associated with cancer progression.

### IL-17A inhibits miR-23b expression through activating NF-κB

**Figure 5:**
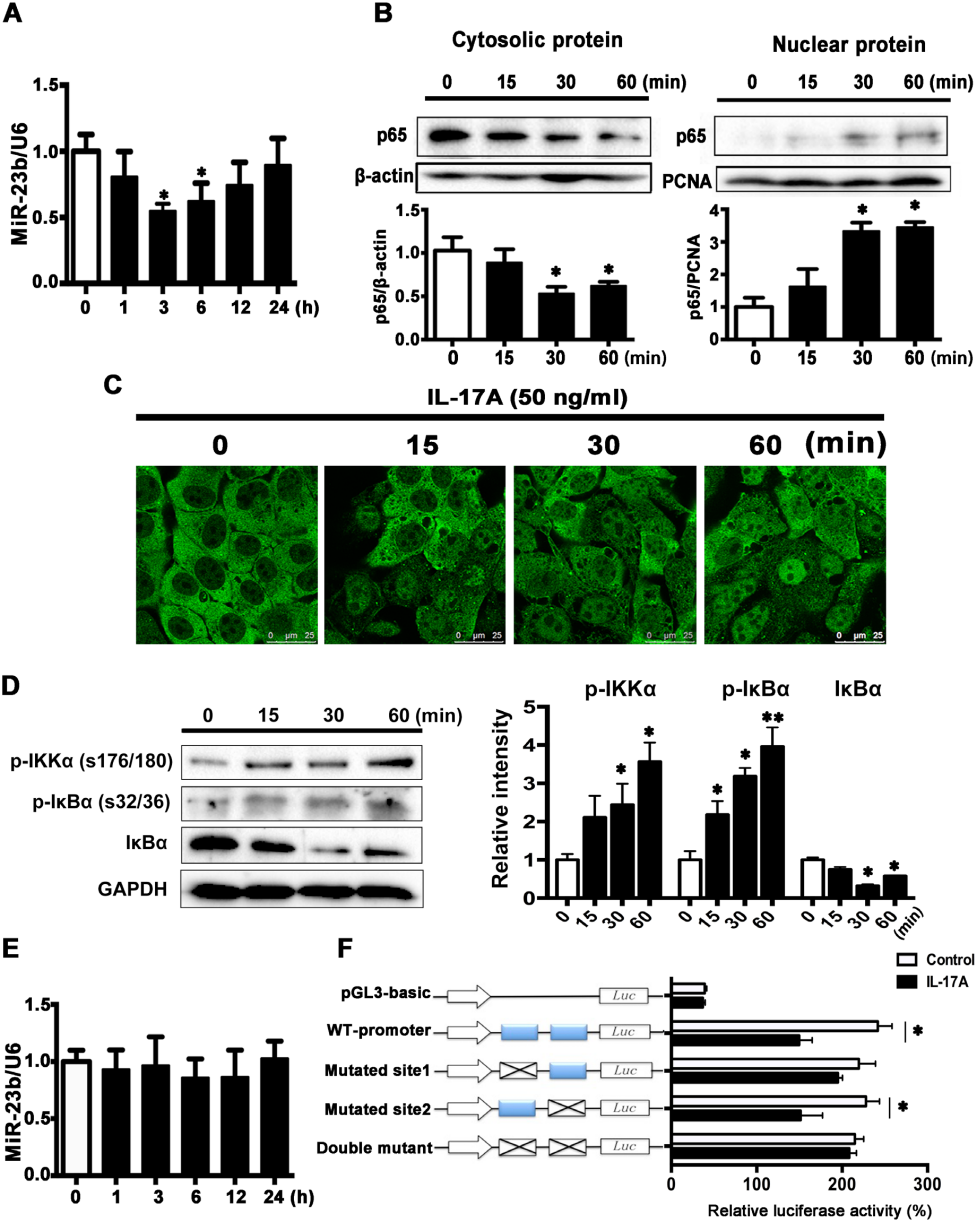
IL-17A downregulates miR-23b expression through activating NF-κB **A**. MiR-23b was decreased after IL-17A (50 ng/ml) treatment. **B**. IL-17A (50 ng/ml) caused a decrease in the amount of p65 protein in the cytoplasm combined with an increase of p65 protein in the nuclear at indicated time points. **C**. Represented immunofluorescent images showing that IL-17A induced the nuclear translocation of p65. Scale bars: 25 μm. **D**. Western blot analysis of p-IKKα (Ser176/178), p-IκBα (Ser32/36), and IκBα proteins after IL-17A treatment (50 ng/ml) in SCC15 cells. Histogram showing the quantitative analysis of those three protein levels was presented in the right panel. **E**. Preincubation of SCC15 cells with pyrrolidine dithiocarbamic acid (PDTC, 50 μM) for 30 min, IL-17A-induced miR-23b downregulation was abolished. **F**. SCC15 cells transfected with luciferase reporter plasmids of pGL3-basic vector, wild-type miR-23b promoter, or promoter with mutated NF-κB binding sites (Mutated site 1, mutated site 2, and double mutant constructs), then left untreated or stimulated with IL-17A for 6 h. Results were showed relative to renilla luciferase activity. Values were presented as mean ± SEM from three independent experiments. *P < 0.05 compared with the untreated cells.

Next, we explored the direct effect of IL-17A on miR-23b expression in SCC15 cells. As shown in Figure 5A, miR-23b expression was decreased in IL-17A-treated cells at 3 and 6 h and returned to baseline at 24 h. NF-κB was a crucial target of IL-17A signaling pathway in many types of cells [31–33] and it had been reported to function as a tumor promoter in inflammation-associated cancer [34]. Western blot analysis showed that IL-17A increased the protein level of p65 in the nuclear fraction and decreased it in the cytosolic fraction (Figure 5B). Immunofluorescence images further confirmed that IL-17A increased NF-κB content in nuclear (Figure 5C). Furthermore, IL-17A significantly increased the levels of phospho-IKKα (p-IKKα, Ser176/178) and phospho-IκBα (p-IκBα, Ser32/36), the upstream signaling components of NF-κB, whereas suppressed IκBα expression (Figure 5D). After pretreated SCC15 cells with 25 μM ammonium pyrrolidinedithiocarbamate (PDTC), an inhibitor of NF-κB, IL-17A-induced miR-23b downregulation was abolished (Figure 5E).

To reveal the exact role of NF-κB in miR-23b expression, we screened the human miR-23b promoter sequence and found two putative NF-κB binding sites: site 1 at -394 to -385 bp and site 2 at -366 to -356 bp relative to the transcription start site of miR-23b. We constructed the luciferase reporters containing either the wild-type miR-23b promoter or the miR-23b promoter with mutated NF-κB binding sites and transfected them into SCC15 cells. IL-17A incubation for 6 h significantly repressed miR-23b transcription activity in SCC15 cells transfected wild-type miR-23b promoter. The repression effects of IL-17A were abolished in SCC15 cells transfected mutated site 1 (-394 to -385 bp mutated) and double-mutated construct, whereas decreased luciferase activity also presented in SCC15 cells transfected mutated site 2 (-366 to -356 bp mutated) construct (Figure 5F). These data suggest that NF-κB inhibits miR-23b transcription by binding to its promoter region directly and IL-17A induced miR-23b transcription repression through NF-κB pathway.

### Overexpression of miR-23b decreases and depletion of miR-23b increases migration and invasion abilities of SCC15 cells

**Figure 6:**
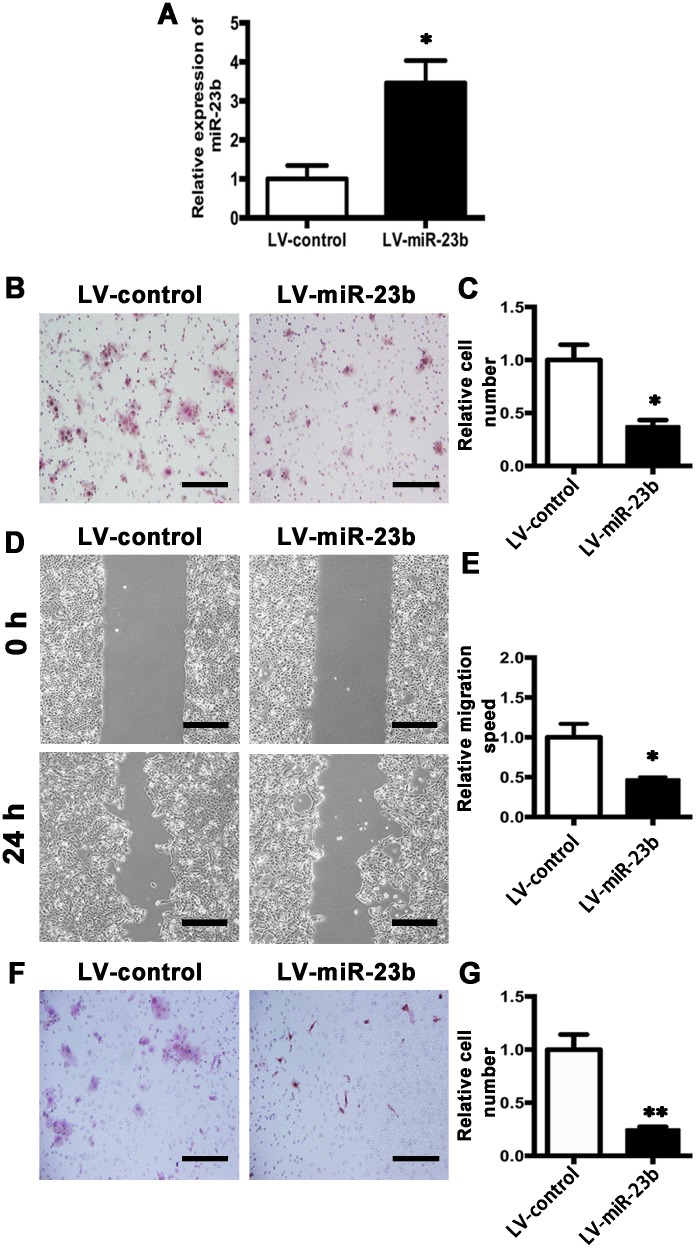
MiR-23b overexpression inhibits migration and invasion abilities of SCC15 cells **A**. Relative miR-23b expression of LV-control and LV-miR-23b cells. **B**. Representative images of cell migration at 24 h for LV-control and LV-miR-23b cells, respectively. **C**. Histogram showing cell migration expressed as relative number of the whole migratory cells on the bottom side of the membrane. **D**. Confluent cells of LV-control and LV-miR-23b were scratched and cell migratory behavior was captured. **E**. Histogram showing cell migration expressed as relative wound edges movement. **F**. Representative images of LV-control and LV-miR-23b cell invasion at 24 h. **G**. Histogram showing cell invasion expressed as relative number of the whole invaded cells on the bottom side of the membrane. Values were presented as mean ± SEM from three independent experiments. Scale bars: 200 μm, *P < 0.05 and **P < 0.01 compared with the LV-control cells.

To elucidate the exact role of miR-23b expression in biological behavior of SCC15 cells, miR-23b stable overexpression cell line was generated by infection of miR-23b lentivirus (LV-miR-23b) and the corresponding control cell was generated by infection of control virus (LV-control) (Figure 6A). Compared with the LV-control cells, the numbers of LV-miR-23b cells moved to the lower chamber were significantly decreased (Figure 6B and 6C). Wound-healing assay confirmed the same phenomenon (Figure 6D and 6E). Furthermore, invasion assay showed that the invasive ability of SCC15 cells was substantially reduced by miR-23b overexpression (Figure 6F and 6G). These data indicate that miR-23b overexpression suppresses the migration and invasion of tongue cancer cells.

**Figure 7:**
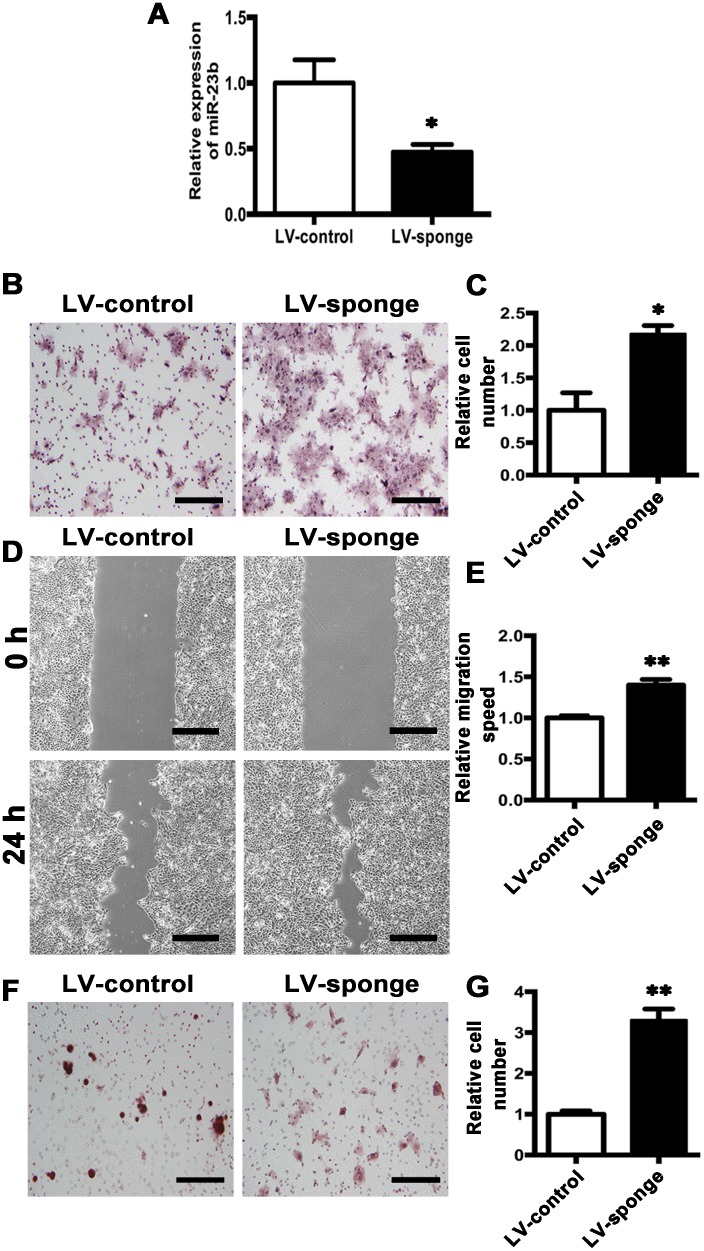
Knockdown of MiR-23b promotes migration and invasion abilities of SCC15 cells **A**. Relative miR-23b expression of LV-control and LV-sponge cells. **B**. Representative images of cell migration at 24 h for LV-control and LV-sponge cells, respectively. **C**. Histogram showing cell migration expressed as relative number of the whole migratory cells on the bottom side of the membrane. **D**. Representative images of wound-healing assay for LV-control and LV-sponge cells at 0 h and 24 h. **E**. Histogram showing cell migration expressed as relative wound edges movement. **F**. Representative images of LV-control and LV-sponge cell invasion at 24 h. **G**. Histogram showing cell invasion expressed as relative number of the whole invaded cells on the bottom side of the membrane. Values were presented as mean ± SEM from three independent experiments. Scale bars: 200 μm, *P < 0.05 and **P < 0.01 compared with the LV-control cells.

We further depleted miR-23b expression by transfection of either anti-miR-23b (LV-sponge) or control virus (LV-control) (Figure 7A). Transwell assay showed that the numbers of LV-sponge cells migrated into the lower chamber were significantly increased than those in LV-control cells. (Figure 7B and 7C) Wound-healing assay further confirmed the results (Figure 7D and 7E). Moreover, invasion assay revealed that downregulation of miR-23b enhanced cell invasion (Figure 7F and 7G). Hence, the results demonstrate that the inhibition of miR-23b expression promotes tongue cancer cell migration and invasion.

### MiR-23b directly targets versican mRNA 3’UTR

**Figure 8:**
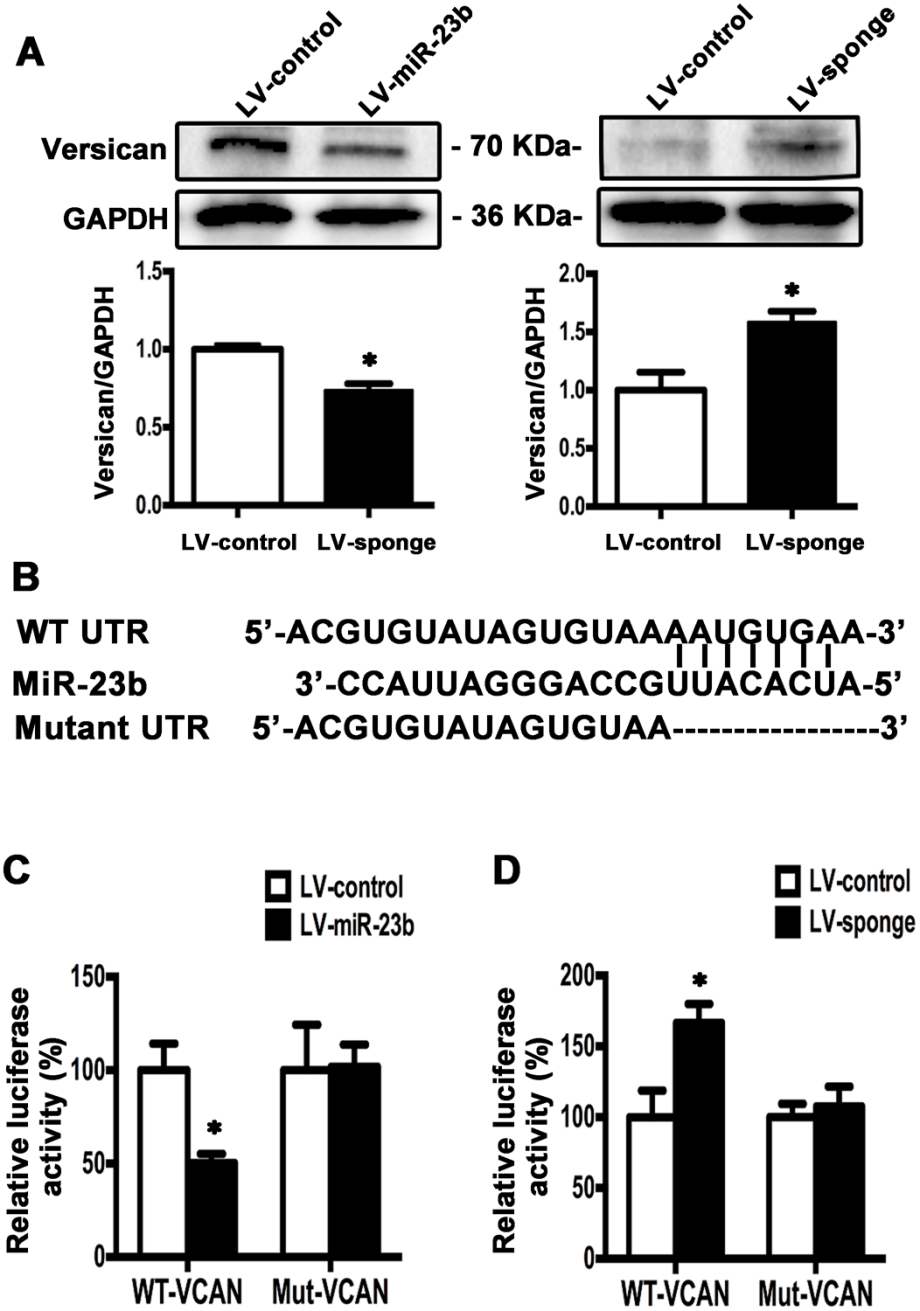
Versican is the downstream target of miR-23b in SCC15 cells **A**. Western blot analysis showed that versican level was decreased in LV-miR-23b cells compared with LV-sponge cells. **B**. WT- and mutated-miR-23b recognition sites of versican 3’UTR region were shown. **C**. Relative luciferase activities were measured 48 h after transfection of WT-VCAN or Mut-VCAN reporters into LV-miR-23b and LV-control cells. **D**. Relative luciferase activities were measured 48 h after transfection of WT-VCAN or Mut-VCAN reporters into LV-sponge and LV-control cells. Values were presented as mean ± SEM from three independent experiments. *P < 0.05 compared with the LV-control cells.

In order to explore the possible target molecule of miR-23b in TSCC cells, we searched the online miRNA database (TargetScan, www.targetscan.org) and found that versican was a potential target of miR-23b with conserved binding site in the 1767 to 1774 bp of 3’UTR region. Western blot analysis showed that overexpression of miR-23b decreased, whereas inhibition of miR-23b increased versican levels compared with the controls (Figure 8A). To further investigate the putative binding sites of miR-23b in versican, we constructed two types of firefly luciferase reporter vectors containing either the wild-type 3’UTR (WT-VCAN) or the mutated 3’UTR region (Mut-VCAN) and conducted reporter assays using LV-miR-23b or LV-sponge cells (Figure 8B). Results showed that overexpression of miR-23b reduced the luciferase activity in WT-VCAN cells, whereas no significant difference was found between LV-control and Mut-VCAN cells (Figure 8C). On the other hand, downregulation of miR-23b increased the luciferase activity in cells transfected with WT-VCAN and no significant difference was detected when the reporter construct was mutated (Figure 8D). These results demonstrate that miR-23b interacts with versican mRNA at the 3’UTR binding site and inhibits versican expression.

### Versican is overexpressed in TSCC specimens and IL-17A upregulates versican expression

**Figure 9:**
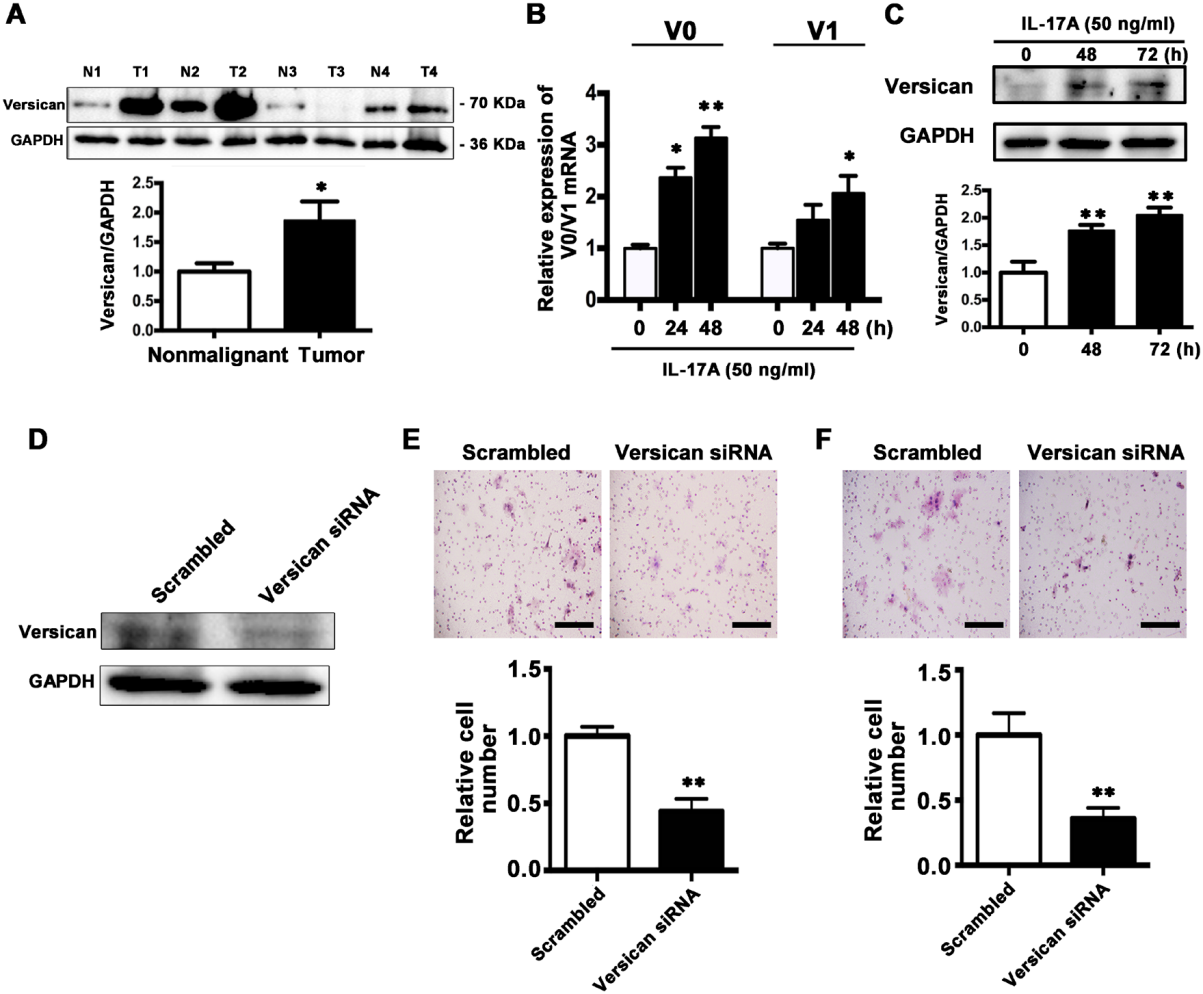
IL-17A promotes versican expression and downregulation of versican inhibits migration and invasion abilities of SCC15 cells **A**. Representative western blot analysis of versican V0/V1 proteins in TSCC tissues and their corresponding controls from 4 patients were shown in the upper panel. Histogram showing the quantitative analysis of versican protein levels based on western blot analysis of 20 pairs of TSCC and paracancer tissues. **B**. Treatment of SCC15 cells with IL-17A (50 ng/ml) at 24 h and 48 h, V0 and V1 mRNA levels were elevated. **C**. Representative western blot analysis of elevated versican levels after treatment of SCC15 cells with IL-17A (50 ng/ml) at 48 h and 72 h. **D**. 100 nM versican siRNA or 100 nM scramble siRNA were transfected to SCC15 cells and the proteins were harvested after 48 h. The expression of versican was analyzed by western blot analysis. **E**. Representative images of cell migration after versican downregulation were shown in the upper panel. Histogram showing cell migration expressed as relative number of the whole migratory cells on the bottom side of the membrane. **F**. Representative images of cell invasion after versican downregulation were shown in the upper panel. Histogram showing cell invasion expressed as relative number of the whole invaded cells on the bottom side of the membrane. Values were presented as mean ± SEM from three independent experiments. Scale bars: 200 μm, *P < 0.05 and **P < 0.01 compared with the untreated cells.

Versican is a secreted proteoglycan protein with diverse functions that can promote tumor progression and its high expression is reported to be associated with unfavorable prognosis [35–37]. Next we examined the V0 and V1 isoforms of versican, the main isoforms expressed among different cancer types, in TSCC tissues. By comparing versican levels in 20 paired TSCC and corresponding paracancer specimens, we found that it was increased in TSCC tissues (16/20, Figure 9A).

We further explored the effect of IL-17A on versican expression. After treatment with IL-17A (50 ng/ml) for 48 and 72 h, the mRNA and protein levels of both V0 and V1 versican were increased (Figure 9B and 9C). These data suggest that IL-17A can upregulate versican expression in SCC15 cells.

### Downregulation of versican inhibits the migration and invasion of SCC15 cells *in vitro*

To reveal the role of versican in cell migration and invasion, SCC15 cells were transfected with either the versican-specific siRNA or the corresponding scrambled siRNA. Western Blot analysis confirmed that versican expression was decreased by 60% after siRNA transfection (Figure 9D). Transwell assay revealed that reduced versican expression significantly inhibited cell migration (Figure 9E). Invasion assay showed that the numbers of cells moved to the lower surface of the membrane were significantly decreased in versican siRNA group (Figure 9F). The results indicate that versican is tumor-promoting protein, and inhibition of versican expression suppresses the migration and invasion ability of SCC15 cells.

## DISCUSSION

In the present study, we demonstrate that the levels of IL-17A in serum and tumor samples are significantly increased in TSCC patients and positively correlate with lymph node metastasis and tumor clinical stages of TSCC. Exogenous IL-17A directly enhances the migration and invasive abilities of SCC15 cells *in vitro*. Moreover, we identify that increased IL-17A is associated with aggressive phenotype of TSCC through miR-23b/versican pathway. Our results provide a novel mechanism that IL-17A in TSCC microenvironment promotes TSCC progression.

Tumor microenvironment and associated key signaling pathways are various depending on tumor types and tissues, thus understanding this diversity between different tumor entities is essential to work out specific therapies [1]. As one of the most crucial proinflammatory cytokines, IL-17A plays an important role in host defense against pathogen infection by stimulating cytokines (TNF-α, GM-CSF, etc) and chemokines (CXCL1, CXCL2, IL-8, etc) expression leading to neutrophil expansion and chemotaxis [2]. Meanwhile, IL-17A is confirmed to be elevated in autoimmune disorders including rheumatoid arthritis and multiple sclerosis, which accelerates disease progression through the induced inflammation [38]. Recently, the role of IL-17A has been investigated in several cancer types. The levels of serum IL-17A are elevated in gastric and bladder cancer patients [39, 40]. T helper 17 cells and IL-17 levels are increased in the peripheral blood of head and neck squamous cell carcinoma patients [41]. However, IL-17A enhances cytotoxic T lymphocytes response resulting in tumor suppression [14, 42]. In breast cancer, higher expression of IL-17 is linked with greater probability for recurrence, greater chemotherapy resistance (to docetaxel), shorter disease free survival, and poor prognosis [43], which suggests that local IL-17A might be used to monitor disease progression and response to therapy. In the present study, we characterized the expression and distribution of IL-17A and its receptor in human tongue tissue. More importantly, we demonstrated that circulating level of IL-17A as well as the contents of IL-17A and IL-17RA in local cancer tissues were significantly increased in TSCC patients. The elevated serum and local IL-17A levels were positively correlated with lymph node metastasis and advanced clinical stages of TSCC patients. Besides, lower IL-17A expression was mainly exists in the early stage TSCC patients. These results suggest that IL-17A has a potential role in the carcinogenesis and progression of TSCC and elevated serum IL-17A level might be a new biomarker for predicting an aggressive phenotype of TSCC.

Despite the positive correlation between IL-17A and TSCC, the exact role of IL-17A in progression of cancer remains unclear. In hepatocellular carcinoma and fibrosarcoma, IL-17A promotes tumor progression through potentiating angiogenesis and metastasis [11, 44]. Here, we found that exogenous IL-17A promoted the migration and invasion of SCC15 cells, but had no effect on their proliferation *in vitro*, which suggests that the elevated IL-17A in tumor microenvironment may exert a direct pro-metastatic role in tumor progression.

We then explored the underlying mechanism in the pro-metastatic effect mediated by IL-17A. Previous studies indicate that IL-17 contributes to autoimmune pathogenesis by suppressing miR-23b expression [28]. Here, we identified that miR-23b was downregulated in 93.7% TSCC tissues. A significant inverse correlation between the increase of IL-17A and decrease of miR-23b was observed in the tested tumors, specifically for those with cervical metastasis and advanced stages. Moreover, NF-κB inhibited miR-23b transcription by directly binding to its promoter region. IL-17A promoted p65 into the nuclear and enhancing the levels of p-IKKα and p-IκBα, whereas inhibiting NF-κB abolished IL-17A induced miR-23b downregulation. These data indicate that IL-17A downregulated miR-23b expression through activating NF-κB. The concomitant IL-17A increase and miR-23b decrease might be crucial to contribute to a progressive phenotype of TSCC.

MiR-23b has been reported to be dysregulated in certain cancer types and act as tumor-suppressor or oncogenes [25, 26, 45]. MiR-23b inhibits migration and proliferation of hepatocellular cancer through suppressing urokinase-type plasminogen activator (uPA) and c-met [26]. MiR-23b is decreased in prostate cancer and overexpression of miR-23b inhibits the migration and invasion of prostate cancer cells [45]. In the present study, we confirmed that overexpression of miR-23b inhibited, while knockdown of it enhanced the migration and invasion abilities of SCC15 cells. These findings indicate that miR-23b plays crucial roles in IL-17A mediated TSCC metastasis and IL-17A promotes migration and invasion of tongue cancer cells through inhibiting miR-23b expression. Recent evidence reports that injection of synthetic antitumor miRNAs successfully inhibits tumor metastasis in animal models [46], which sheds light on the exploitation of miR-23b based therapies to treat metastatic TSCC with IL-17A upregulation.

Versican, a chondroitin sulfate proteoglycan, is the major component of extracellular matrix ubiquitously expressed in almost all tissues and regulates varieties of cellular processes including cell proliferation, migration and invasion through its chondroitin and dermatan sulfate side chains and the G1 and G3 domains [47–49]. Accumulating evidence indicates that increased versican expression is associated with unfavorable prognosis for many cancers [50, 51]. Overexpression of versican promotes cell survival and apoptotic resistance combined with downregulated Fas protein levels [52, 53]. Versican can also activate resident fibroblasts and endothelial cells in tumor stroma through toll-like receptors (TLR) 2 and TLR6, resulting in enhanced neovascularization [54]. Moreover, elevated versican is reported to increase cancer cell motility and invasion [55, 56]. Expression of versican is regulated by multiple cytokines [57–59]. TGF-β2 enhances versican expression in fibrosarcoma and osteosarcoma cells [57] and IL-1β increases versican levels in lung fibroblasts [59]. Here, we found that versican level was elevated in TSCC tissues and IL-17A upregulated versican expression in SCC15 cells both in mRNA and protein levels. Moreover, luciferase activity assay confirmed that miR-23b directly targeted the 3’UTR region of versican and suppressed versican expression. Knockdown of veisican inhibited SCC15 cell migration and invasion. These results indicate that IL-17A increases versican expression, at least in part, through regulating miR-23b. Versican functions as a tumor promoter by regulating cell-extracellular matrix interaction.

In conclusion, our study demonstrated that IL-17A is an important proinflammatory cytokine to accelerate TSCC progression. The elevated IL-17A promotes TSCC cell migration and invasion through modulating miR-23b/versican exprssion. These findings provide a new insight into the biological function of IL-17A in tumor immunity. Elucidating the role of the IL-17A/miR-23b/versican pathway might enlarge our understanding of the interaction between inflammatory microenvironment and cancer progression, which can be exploited for potential immunotherapeutic and diagnostic target in TSCC.

## MATERIALS AND METHODS

### Patients and samples

The study was approved by the Ethics Committee of Peking University School and Hospital of Stomatology (Approval number PKUSSIRB-2013009) and all the participants signed the informed consent before blood and tissue collection. 76 patients underwent surgery for pathologically confirmed TSCC at Peking University School and Hospital of Stomatology between October, 2012 and June, 2015 were enrolled in this study. None of the patients had received any anti-cancer therapy before sample collection. In addition, 15 age-matched healthy controls were recruited as volunteers.

Fasting blood samples were collected from patients (n = 25) and controls (n = 15) in the morning. Serum was separated immediately by centrifugation and stored at -80°C until further analysis. Part of surgically resected tumor tissues and adjacent normal tissues (at least 2 cm away from tumor margins) were frozen in liquid nitrogen at once and stored at -80°C. The remaining parts were paraffin-embedded for histopathological analysis. Tumor clinical stage and histological grading were classified based on the 7^th^ edition of the TNM classification of American Joint Committee on Cancer [29].

### Enzyme linked immunosorbent assay (ELISA)

Concentration of cytokines in the serum of TSCC patients and healthy controls were measured using commercial ELISA kits (eBioscience, CA, USA). All assays were performed in duplicate.

### Immunohistochemistry

Consecutive 5 μm sections were incubated with primary antibody against IL-17A (Bioworld, MN, USA) at 4°C overnight followed by HRP-conjugated secondary antibodies (Zhongshan Goldenbridge, Beijing, China). Five randomly chosen fields of each section were used to evaluate the image staining by using Image J software (NIH, MD, USA) and LEICA 550IW system as previously described [60].

### Cell culture

SCC15 cell line was purchased from American Type Culture Collection (ATCC, VA, USA) and cultured at 37°C with 5% CO_2_ atmosphere in DMEM/F12 (1:1 mixture) medium containing 10% fetal bovine serum (FBS). All reagents were purchased from Sigma-Aldrich (Sigma-Aldrich, MO, USA).

### Quantitative real-time PCR

Total RNA was isolated from tissues and cells by using TRIzol reagent (Thermo Fisher Scientific, MA, USA) according to the manufacturer's instruction. The primer sequences are shown in Table 2. Real-time PCR was performed on a PikoReal Real-Time PCR System (Thermo Fisher Scientific) by utilizing DyNAmo^TM^ ColorFlash SYBR Green qPCR kit (Thermo Fisher Scientific).

### Western blot analysis

Tissues and cells were homogenized in lysis buffer (Thermo Fisher Scientific) with protease inhibitors (Roche, Basel, Switzerland). After centrifugation, the supernatants were collected and the protein concentration was measured. Equal amounts of proteins (30 μg) were separated on SDS-PAGE and transferred to polyvinylidene difluoride membrane. After blocking with 5% non-fat milk, the membrane was probed with primary antibodies at 4°C overnight followed by HRP-conjugated secondary antibodies (Zhongshan Goldenbridge). Immunoreactive bands were visualized with enhanced chemiluminescence (Thermo Fisher Scientific). The densities of bands were quantified by Image J software (NIH).

### Isolation of nuclear and cytosolic protein

Nuclear and cytosolic protein extracts were separated by a commercially available separation kit (Thermo Fisher Scientific) according to the manufacture's instruction.

### Proliferation assays

SCC15 cells were grown on 96-well plates (5 × 10^3^ cells at 100 μl/well) and cultured with 5% FBS with or without IL-17A (12.5-100 ng/ml; R&D systems, MN, USA) for 48 h. The proliferation was evaluated by a colorimetric assay using the CellTiter 96 AQueous One Solution (MTS) reagent (Promega, WI, USA). All assays were conducted in triplicate.

### Migration assay

Cell migration was measured by wound-healing and transwell assay. For wound-healing assay, cells were grown to confluence in 6-well plates and a scratched wound was made by a sterile tip in the layer. Six randomly chosen regions were captured at 0 and 24 h and the relative migration speed was measured by the mean linear movement speed of wound edge. For transwell assay, cells were seeded to a 24-well cell culture chamber (8 μm pore size; Corning, NY, USA) in 100 μl serum-free medium. After 24 h incubation, cells on the lower side of the membrane were fixed in 95% ethanol and stained with hematoxylin and eosin. The whole membrane was photographed and the cells on the membranes were counted. All experiments were performed in triplicate.

### Invasion assay

Invasion assay was performed using a 24-well cell culture chamber precoated with Matrigel (Corning). The following steps were the same as transwell assay.

### Immunofluorescence staining

SCC15 cells were cultured on glass coverslips, fixed with 4% paraformaldehyde, and incubated with 5% bovine serum albumin in phosphate buffed saline. Then, they were incubated with the primary antibody against p65 (Abcam, Cambridge, UK) overnight at 4°C and followed by secondary Alexa Fluro 488 goat anti-rabbit IgG (Zhongshan Goldenbridge) for 2 h at 37°C. Nuclei were stained with 4, 6-diamidino-2-phenylindole (DAPI) (Sigma-Aldrich). Fluorescence images were acquired on a confocal microscope (Leica TCS SP5, Wetzlar, Germany).

### Overexpression and inhibition of miR-23b

MiR-23b and ~150 bp of flanking sequence was amplified from human genomic DNA and cloned into *p*EASY^®^-T3 Cloning Vector (Transgen Biotech, Beijng, China). After sequencing confirmation, the fragment was digested with *Eco*RI and *Bam*HI and cloned into lentiviral vector pCDH-CMV-MCS-EF1-copGFP (LV-miR-23b; System Bioscience, CA, USA). Lentiviral miR-23b sponge (LV-sponge) was constructed by Hanbio Co. LTD (Shanghai, China). Virus was created and infected targeted cells using a polybrene reagent (Merck Millipore, Darnstadt, Germany) according to manufacture's instruction.

### Genetation of miR-23b promoter fragments and its mutant

A human miR-23b (-1200 to -200) promoter fragment was amplified by PCR from genomic DNA of SCC15 cells and cloned into the pGL3-basic vector (Promega) upstream of the firefly luciferase. Mutated promoter products were constructed by performing overlap extension PCR through two pairs of primers without the putative binding sequences. All the plasmids were confirmed by sequencing before use.

### Generation of 3’UTR of versican and its mutant

The 3’UTR of versican (1813 bp) was amplified using PCR from complementary DNA synthesized from total RNA. The used primers contained the following restriction sites: forward, 5’-ACTAGTCAAAGTCCTAACTTCCTGTGCCTTT-3’ (SpeI); reverse, 5’-AAGCTTGTGTAGTAAAAGAAGGATTTTAGGTTTC-3’ (HindIII). The PCR product was ligated to the pMIR-REPORT vector (Thermo Fisher Scientific) downstream of the open reading frame of luciferase to generate the wild type versican reporter (WT-VCAN).

Mutated 3’UTR product was constructed by performing overlap extension PCR through two pairs of primers without the putative binding sequence. The mutant was cloned into the pMIR-REPORT vector to generate the mutant versican reporter (Mut-VCAN). All plasmids were verified by sequencing before use.

### Luciferase reporter assay

Dual-Luciferase reporter assays were performed according to the manufacture's instruction (Promega). Relative luciferase activity represents firefly luciferase normalized against renilla luciferase activity.

### Knockdown of versican

SCC15 cells were cultured to 70% confluence and then transfected with siRNA through using Lipofectamine 2000 reagent (Thermo Fisher Scientific) according to manufacture's instruction. The siRNA of versican and non-specific negative control were purchased from Sigma-Aldrich: versican (SASI_Hs02_00337487).

### Statistical analysis

Data are presented as mean ± standard error of the mean (SEM). Statistical analyses were performed using SPSS Statistics version 20.0 (SPSS, Chicago, IL, USA). Differences were determined by a Student's unpaired *t*-test for two groups or an ANOVA with multiple groups, followed by Turkey *post hoc* testing. Correlation between IL-17A expression and miR-23b levels was analyzed using Pearson's coefficient correlation analysis. *P* < 0.05 was considered statistically significant.
